# Safety and Feasibility of In Situ Fenestration in the Aortic Arch: A Prospective Single-Center Observational Cohort Study

**DOI:** 10.3390/jcm15135267

**Published:** 2026-07-06

**Authors:** Ralf Kolvenbach, Chang Shu, Elisa R. Lica

**Affiliations:** 1 Sana Hospital Group, Department of Vascular Surgery, 40625 Duesseldorf, Germany; ralu2407@yahoo.com; 2 State Key Laboratory of Cardiovascular Disease, Fuwai Hospital, Beijing 100037, China; changshu@vip.126.com; 3 National Center for Cardiovascular Diseases, Beijing 100037, China; 4 Chinese Academy of Medical Sciences, Peking Union Medical College, Beijing 100037, China

**Keywords:** aortic arch repair, endovascular intervention, in situ fenestration, TEVAR, thoracic aortic aneurysm

## Abstract

**Background:** Thoracic endovascular aortic repair (TEVAR) is an established minimally invasive approach for selected aortic arch pathologies; however, it is associated with risks including stroke and spinal cord ischemia. Revascularization techniques, such as in situ fenestration (ISF), play a critical role in preserving supra-aortic branch perfusion and reducing neurological complications. **Methods:** This prospective, single-center observational cohort study enrolled 74 consecutive patients undergoing TEVAR with ISF between October 2017 and September 2023. Data collected included demographics, lesion morphology, procedural details, and clinical outcomes. The primary endpoint was procedural technical success; secondary endpoints included 30-day complications, reintervention rate, and all-cause mortality. **Results:** Technical success was achieved in 100% of cases (74/74; 95% CI: 95.2–100.0%), defined as successful fenestration creation, patent bridging stent graft without kinking or embolization, absence of Type I or III endoleak on completion angiography, and restored antegrade branch flow. Physician-modified fenestration was combined with ISF-thoracic endovascular aortic repair (TEVAR) in 28.4% (21/74) of cases. At 30-day follow-up, 30-day clinical success (freedom from mortality, reintervention, and procedure-related complications) was achieved in 87.8% (65/74) of patients. No mortality was recorded at 30 days. Treatment-related complications included subclavian branch thrombosis (*n* = 1, 1.4%), transient ischemic attack (*n* = 1, 1.4%), and endoleaks (*n* = 7, 9.5%; including Type Ia, Type II, and Type III), with reintervention required in 6 patients (8.1%) during the follow-up period. Beyond 30 days, three late deaths were documented: one aorta-related death (aneurysm rupture at 9 months), one neurological death (ischemic stroke at 13 months), and one cardiovascular death (myocardial infarction at 60 days post-procedure), yielding a late all-cause mortality rate of 4.1% (3/74). **Conclusions:** ISF-TEVAR demonstrated a high procedural technical success rate and a low 30-day complication and mortality profile in this single-center prospective series of selected patients treated at an experienced center. These early and mid-term results are encouraging; however, given the single-center, non-comparative design and limited standardized follow-up, broader conclusions regarding durability and comparative effectiveness remain premature. Larger multicenter prospective studies with standardized long-term imaging follow-up are warranted.

## 1. Introduction

Thoracic endovascular aortic repair (TEVAR) is a minimally invasive procedure indicated for conditions such as aneurysms involving the descending aorta and/or the aortic arch [1], Type B [2], or Type non-A non-B [3] aortic dissections, penetrating atherosclerotic ulcers, and intramural hematomas [4]. TEVAR has become a widely used alternative to open surgical repair for many elective thoracic aortic pathologies, providing a rapid and reproducible revascularization strategy with favorable early outcomes in prior reports [4,5]. A systematic review reported a significantly lower 30-day in-hospital mortality rate with TEVAR compared with open repair, with a pooled odds ratio of 0.54 (*p* < 0.00001) [6]. The 180-day mortality rate was higher in patients who underwent open surgical repair (23.9%; 21.5–26.2%) than in those who received TEVAR (10.4%; 9.2–11.6%) [7]. Although open surgery reduces the risk of reintervention and long-term risk of death, TEVAR provides an early survival advantage that persists for at least 9 years, resulting in an overall survival benefit [7].

The aortic arch presents a unique anatomical and hemodynamic challenge for endovascular repair. Accurate landing zone classification is fundamental to procedural planning: Zone 0 encompasses the ascending aorta proximal to the innominate artery, Zone 1 lies between the innominate and left common carotid arteries, Zone 2 between the left common carotid and left subclavian arteries, and Zone 3 begins distal to the left subclavian artery. Proximal extension of stent graft coverage into Zones 0–2 necessitates deliberate strategies to maintain patency of the supra-aortic branches, as inadvertent occlusion of these vessels, particularly the left subclavian artery (LSA), carries substantial risk of neurological morbidity, including ischemic stroke, posterior circulation events, and spinal cord ischemia via compromise of the anterior spinal artery territory.

Neurological complications are among the most common concerns following TEVAR [8]. Several procedural risks are associated with TEVAR, including silent brain infarction, often caused by cerebral embolization during aortic arch instrumentation [5,9]. The Stroke from Thoracic Endovascular Procedures registry findings show that silent brain infarction occurs in approximately 50% of cases after endovascular treatment of the aortic arch. Urgent procedures and deployment in Zone 0–1 were identified as risk factors for these [9]. Moreover, the risk of perioperative stroke increases with the complexity of endovascular aneurysm repair (EVAR) and TEVAR procedures [10]. In an 8-year study by Swerdlow et al., the in-hospital stroke rate was 0.2% after EVAR, 0.9% after complex EVAR, and 2.6% after TEVAR [11]. Strokes secondary to spinal cord ischemia or left upper extremity ischemia may arise due to the coverage of the LSA in approximately 26–40% of patients undergoing TEVAR [5]. Additionally, patient-related risk factors for ischemia include advanced age and large aneurysms [5].

**Figure 1:**
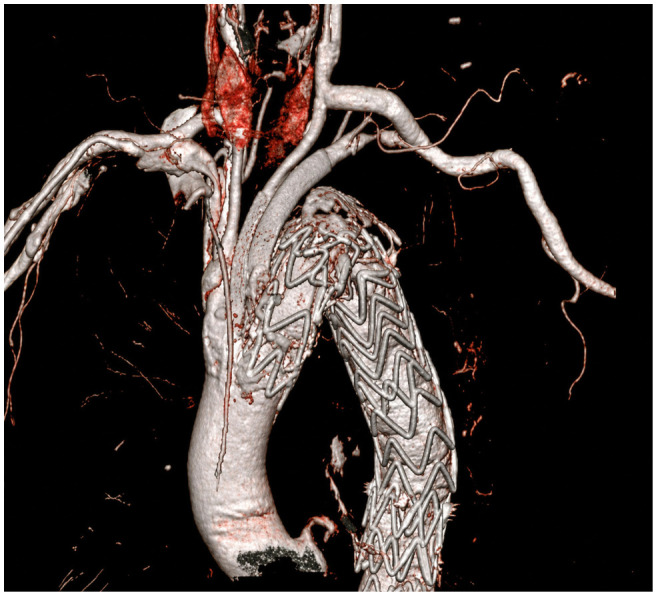
Three-dimensional computed tomography angiography demonstrating thoracic endovascular aortic repair with in situ fenestration and supra-aortic branch revascularization of the left subclavian artery.

Revascularization of the supra-aortic branches is crucial to minimizing complications. Among patients who underwent TEVAR, the stroke rate was 2.8% for those with LSA revascularization, while those without LSA revascularization had a stroke rate of 11.8% [12]. Techniques such as in situ fenestration (ISF) (Figure 1), chimney stenting, and branched and fenestrated stent grafts are common methods of revascularization during TEVAR [13]. An ISF is an adaptable and efficient method for LSA revascularization. may avoid the need for custom-manufactured grafts in selected cases [13]. Laser, needle, and radiofrequency ablation are commonly used for ISF [13,14].

Laser ISF is especially valuable in emergency settings, offering surgical precision in patients with complex aortic arch pathologies and high surgical risks [15,16]. Chimney stenting provides good graft patency and low mortality at 1.2% [17]. However, it carries a risk of proximal gutter endoleaks due to incomplete sealing. Custom-made branched and fenestrated stent grafts have limitations, including long manufacturing times and increased stroke risk from excessive arch manipulation [13]. Combining self-radiopaque markers with physician-modified fenestration (PMF) may improve procedural accuracy in 98% of cases [18].

Each of these revascularization strategies entails distinct technical considerations and risk profiles. Chimney (or “snorkel”) stenting, while widely available and applicable to urgent cases, is associated with a reported gutter endoleak rate of 10–20% depending on series and follow-up duration. It is attributable to incomplete apposition between the parallel stent graft and the main aortic endograft. Branched and fenestrated stent grafts, when custom-manufactured, provide anatomically optimized solutions with low endoleak rates. However, they require manufacturing lead times of 4–8 weeks under standard regulatory frameworks, precluding their use in urgent or emergency presentations. On-the-table PMF represents a pragmatic intermediate approach, enabling off-the-shelf devices to be adapted intraoperatively by having fenestrations cut by the operating surgeon. However, this technique demands advanced operator expertise and is subject to regulatory variation across jurisdictions. ISF, by creating the fenestration in situ within the already-deployed endograft, avoids the anatomical and geometric uncertainties inherent to pre-deployment modification. Hence, ISF may facilitate more accurate alignment between the fenestration and the target vessel ostium.

This study aimed to evaluate the feasibility and short-term safety profile of ISF-TEVAR for aortic arch pathologies requiring preservation of the LSA and/or the left common carotid artery (LCCA). The objectives of this study were to assess procedural success, determine the rate of complications following ISF-TEVAR and the reinterventions required, and assess patient survival at 30-day and post-30-day follow-up.

## 2. Materials and Methods

### 2.1. Study Design and Population

This is a prospective, single-center, observational cohort study of nonrandomized patients with aortic arch pathology requiring endovascular intervention. Patients were enrolled consecutively during the study period, and all eligible individuals meeting the inclusion criteria were offered participation without preselective exclusion based on comorbidity burden, thereby reflecting a real-world, all-comers cohort. Both elective and emergency presentations were included; however, given that procedural urgency was not captured as a discrete variable in the institutional database, formal stratified outcome analysis by urgency status could not be performed. The distribution of aortic pathologies across the cohort is characterized. These conditions were not separately analyzed owing to the study’s descriptive, exploratory nature and the lack of sufficient statistical power for meaningful subgroup comparisons.

All patients were managed within a dedicated institutional aortic program. Complex endovascular arch cases were reviewed at a multidisciplinary aortic team meeting prior to elective procedures, incorporating input from vascular surgery, interventional radiology, and cardiac anesthesiology. Emergency cases were triaged by the on-call vascular surgery team with senior oversight. Patients and/or their families were specifically informed of the off-label or customized nature of ISF and PMF procedures, and informed consent explicitly covered the use of physician-modified or on-table-modified endovascular techniques where applicable.

This was a retrospective chart review of routinely collected clinical data. All procedures were performed as part of standard clinical care and not for research purposes. The study protocol, specifically, the retrospective analysis of pre-existing, de-identified clinical data, was reviewed and approved by the Institutional Review Board (IRB approval number: 2.2022) in accordance with the Declaration of Helsinki. The approval covered the analysis of the de-identified clinical data; it did not pertain to any intervention, treatment, or research-specific data collection, as none was undertaken. At the institution, patients provide general written consent upon admission permitting the use of their clinical data for future scientific research, provided personal identification is concealed. All data were fully anonymized prior to analysis, in accordance with institutional data protection policy.

**Figure 2:**
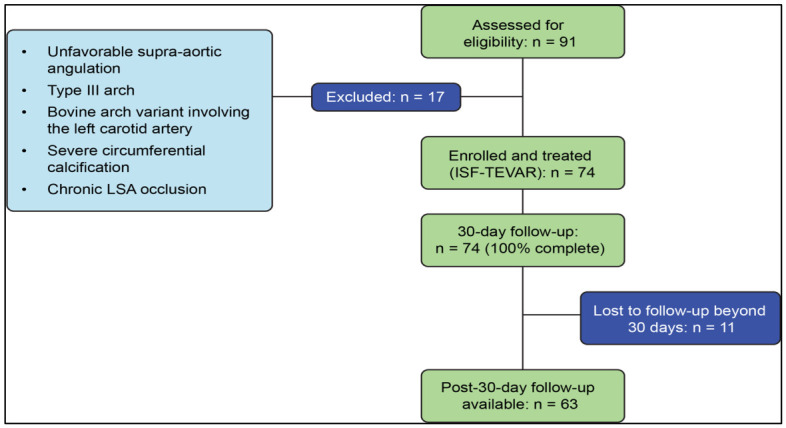
Patient flow diagram. LSA: Left subclavian artery; ISF-TEVAR: In situ fenestration thoracic endovascular aortic repair.

A total of 91 patients were eligible for inclusion (Figure 2). Of these, 17 were excluded owing to anatomical factors, including unfavorable angulation of the supra-aortic vessels or the aortic arch. A total of 74 patients who met the inclusion criteria were enrolled between October 2017 and September 2023 using consecutive sampling. A patient flow diagram illustrating screening, exclusion, enrollment, and follow-up availability is provided as Figure 2. All 74 enrolled patients had complete 30-day follow-up data. Post-30-day follow-up data, based on routine clinical visits and available institutional records, were available for 63 patients (85.1%); 11 patients (14.9%) were lost to follow-up beyond 30 days.

Data collected included demographics, morphologic features of the aortic lesion, details of the intervention and endograft used, and postoperative outcomes during hospitalization and follow-up. A broad eligibility design was implemented, allowing the inclusion of patients irrespective of their comorbidities or baseline characteristics to ensure a diverse and representative study population.

Anatomical suitability for ISF-TEVAR was assessed on the basis of the following morphological criteria: (1) adequate proximal landing zone length of ≥20 mm at Zone 2; (2) the angle of the LSA take-off relative to the aortic centerline was assessed during preoperative planning as part of anatomical suitability criteria (≤60°). This parameter was used for procedural planning and case selection; however, no formal statistical analysis was performed to evaluate its independent association with outcomes; (3) absence of severe circumferential calcification or thrombus at the intended fenestration site; and (4) target vessel diameter ≥5 mm at the ostium to accommodate bridging stent graft placement. Patients with a Type III arch configuration, characterized by a downwardly directed arch with a vertical angle that significantly increases catheter manipulation difficulty and embolization risk, and those with a bovine arch variant involving the left carotid artery were excluded, as these configurations preclude safe and reproducible ISF performance with currently available needle-based systems.

Preoperative patient risk stratification was performed using established scoring systems. Cardiac risk was assessed using the Revised Cardiac Risk Index, and, where applicable, overall surgical risk was estimated using the European System for Cardiac Operative Risk Evaluation to contextualize the cohort’s operative risk profile and justify the endovascular approach over open surgical repair in individual cases.

Inclusion Criteria:

- Patients undergoing elective or emergency TEVAR requiring LSA revascularization, with or without LCCA involvement;
- Isolated LSA revascularization with Zone 2 TEVAR;
- Availability of preoperative computed tomography angiography (CTA) conducted within 6 months before TEVAR.

Exclusion Criteria:

- Patients presenting with supra-aortic anatomical variations;
- LSA revascularization or preservation with Zone 0–1 TEVAR;
- Patients with a Type III arch;
- Patients with a bovine configuration involving the left carotid artery;
- Patients with severe aortic arch calcifications or chronic occlusion of the LSA.

### 2.2. Procedure

All procedures were performed by two senior vascular surgeons (R.K. and C.S.), each with more than 15 years of experience in complex endovascular aortic interventions, supported by dedicated interventional radiology personnel. All procedures were performed in a fully equipped hybrid operating theater with fixed-installation high-resolution fluoroscopy Cios Alpha^®^ mobile C-arm system (Siemens Healthineers, Erlangen, Germany), allowing biplanar imaging when required. General anesthesia was administered in all elective cases; in emergency settings, the anesthetic modality was determined by the attending anesthesiologist on a case-by-case basis, with local anesthesia supplemented by conscious sedation when general anesthesia posed a prohibitive risk.

The study descriptively evaluated the outcomes of ISF-TEVAR, including cases performed with adjunctive or modified techniques, using stents from different manufacturers. No formal comparative statistical analysis of techniques was performed, in keeping with the study’s exploratory, single-arm design. The stents used included the Ankura™ TAA Stent Graft System (Lifetech Scientific Corporation, Shenzhen, China), GORE^®^ Viabahn^®^ Endoprosthesis (W. L. Gore & Associates, Inc., Flagstaff, AZ, USA), Boston Scientific Express™ LD Iliac and Biliary Stent System (Boston Scientific Corporation, Marlborough, MA, USA), Bard Fluency™ Plus Endovascular Stent Graft (Bard Peripheral Vascular, Inc., a subsidiary of Becton, Dickinson and Company, Tempe, AZ, USA), Dynamic^®^ balloon-expandable stent (Biotronik, Berlin, Germany), and the Medtronic Valiant^®^ thoracic stent graft (Medtronic, Minneapolis, MN, USA). Procedural details, including equipment and technique, were recorded for each case.

Intraoperative anticoagulation was administered with unfractionated heparin, targeting an activated clotting time > 250 s, consistent with standard endovascular aortic practice. Postoperative antiplatelet therapy (single antiplatelet agent) was initiated in all patients unless contraindicated. Long-term antithrombotic management was individualized based on indication, bridging stent type, and comorbidity profile. Intraoperative endoleak assessment was performed on completion angiography in all cases; transesophageal echocardiography and intravascular ultrasound were not routinely used, and cone-beam CT was unavailable at this center during the study period.

### 2.3. Preoperative Imaging and Planning

CTA was used to evaluate aortic arch morphology. Datasets were transferred to dedicated 3D vascular planning software (3mensio Vascular^®^, version 10.0, Pie Medical Imaging B.V., Maastricht, The Netherlands; or Aquarius iNtuition^®^, version 4.10, TeraRecon, Inc., Durham, NC, USA) for centerline-based measurements and stent graft sizing. Analysis focused on maximal aortic diameter, shape, extent, and involvement of aortic branches, as visualized on axial, multiplanar, and 3D volume-rendered images. Access feasibility was determined by measuring the inner and outer diameters of the larger iliac or femoral arteries. The minimum access diameter was defined as the narrowest point along this axis. Outer-to-outer wall diameters were obtained at the prospective landing Zone 2. Neck lengths of the aorta were computed by measuring curvatures (inner, outer, and centerline) from the distal edge of the LCCA to the proximal edge of the LSA or aorta. For the LSA, vessel feasibility was assessed using length to the ipsilateral vertebral artery level, diameter, and angulation from the aortic arch.

Anatomical eligibility was assessed using the Ishimaru landing zone classification, with Zone 2 (distal to the left common carotid artery, proximal to the left subclavian artery) serving as the required proximal landing zone for all procedures in this series. Endograft oversizing was calculated relative to the true aortic lumen diameter at the intended landing zone, with a target of 10–20% to ensure adequate radial fixation and sealing without excessive distortion of the graft fabric, a critical factor in preserving fenestration geometry during and after ISF creation. The calculated fenestration diameter was matched to the bridging stent graft diameter, typically selected to be 1–2 mm larger than the ostial diameter of the target branch vessel to ensure adequate sealing at the bridging stent–main body interface. Detailed patient-level quantitative anatomical parameters (individual arch angulation measurements, aortic diameter at the landing zone, and calcification burden scores) were used for operative planning but were not recorded as discrete database variables, precluding systematic tabular reporting. For emergency presentations in which the 6-month CTA criterion could not be met, the most recent available cross-sectional imaging obtained within 72 h of the intervention was used.

### 2.4. Postoperative Imaging

Postoperative CTA was performed within 24–48 h of the index procedure in all cases to assess the technical result and serve as a baseline for follow-up comparison. Scheduled follow-up CTA was planned at 3 and 12 months; however, adherence to this protocol was not uniform across the study period, and imaging follow-up was not standardized for all patients, as acknowledged in Section 5. Endoleak was classified according to the standard five-type classification system; Type I endoleak was defined as flow into the perigraft space at the proximal (Type Ia) or distal (Type Ib) seal zone; Type II as retrograde branch filling; Type III as mid-graft leak; Type IV as graft porosity. Branch stenosis was defined as ≥50% luminal reduction on CTA or duplex ultrasound. Sac enlargement was defined as an increase in the maximum diameter of ≥5 mm relative to the first postoperative CTA. Imaging was reviewed by the operating surgeons; independent radiological adjudication was not performed systematically, which is acknowledged as a limitation.

### 2.5. Vascular Access and Stent Placement

The initial step involved gaining vascular access through the femoral or iliac artery, using a surgical cut-down or a percutaneous technique, as determined to be feasible. Stent selection was individualized for the patient and determined by specific indications for endovascular intervention, lesion location, and vessel diameter. All stent placements were performed under angiographic guidance to ensure accurate positioning and deployment.

### 2.6. ISF Technique

**Figure 3:**
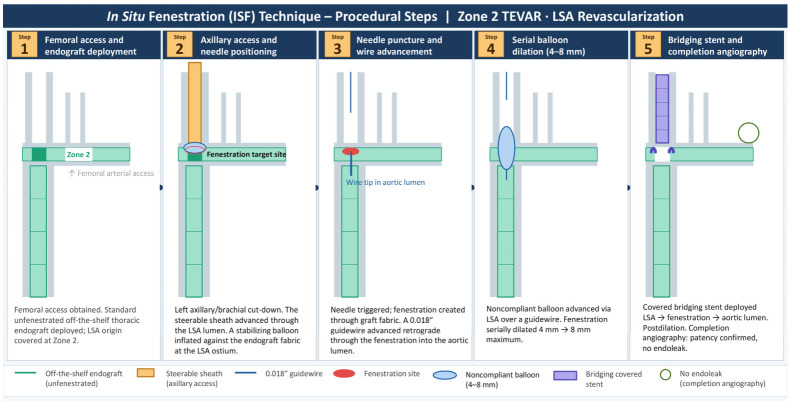
Schematic illustration of the needle-based in situ fenestration (ISF) technique for left subclavian artery (LSA) revascularization during Zone 2 thoracic endovascular aortic repair (TEVAR). Step 1: Deployment of a standard unfenestrated off-the-shelf thoracic endograft in Zone 2, intentionally covering the LSA origin. Step 2: Introduction of a steerable sheath via left axillary/brachial access and positioning at the fenestration target site with stabilization against the endograft fabric at the LSA ostium. Step 3: Needle puncture of the endograft fabric and advancement of a 0.018-inch guidewire through the fenestration into the aortic lumen. Step 4: Serial dilation of the fenestration using a non-compliant balloon (4–8 mm). Step 5: Deployment of a covered bridging stent from the LSA through the fenestration into the aortic lumen, followed by completion angiography confirming patency and absence of endoleak. Color coding: green, thoracic endograft; yellow, steerable sheath; light blue, guidewire; blue, non-compliant balloon; purple, covered bridging stent; red, fenestration site; green circle, absence of endoleak. ISF = in situ fenestration; LSA = left subclavian artery; LCCA = left common carotid artery; TEVAR = thoracic endovascular aortic repair.

A self-centering adjustable needle-based puncture device (FuThrough™ Endovascular Needle System, Lifetech Scientific Corporation, Shenzhen, China; 20 G puncture needle) was introduced via left axillary/brachial access and advanced through the LSA lumen, positioning the device in contact with the outer curvature of the deployed endograft at the intended target vessel ostium. The stabilizing balloon at the tip of the needle device was inflated against the endograft fabric to secure the system and preselect the penetration depth. Once the needle was triggered, a fenestration was created through the graft fabric. A 0.018-inch guidewire was then advanced retrograde through the fenestration into the aortic lumen. The fenestration was progressively dilated using non-compliant balloons, starting at 4 mm and increasing to 8 mm. A bridging stent graft was subsequently deployed to connect the aortic main body and the target branch vessel, and balloon dilatation was performed to optimize the proximal seal. Completion angiography confirmed technical success, vessel patency, and the absence of Type I or Type III endoleak. A procedural schematic is shown in Figure 3.

### 2.7. ISF Combined with the Self-Radiopaque-Guided PMF Technique

In cases requiring multi-vessel arch revascularization (LCCA and/or innominate artery in addition to the LSA), ISF was combined with self-radiopaque-guided PMF. Preprocedural angiography was performed, and fenestration was created under radiographic guidance using radiopaque markers incorporated into the stent graft. Accurate alignment of the fenestration with the target vessel ostium was ensured using radiopaque marker visualization and fluoroscopic monitoring. The stent graft was fully deployed, and a bridging stent was placed through the fenestration to connect the aorta and target branch artery.

### 2.8. Definition of Technical Success

Technical success of ISF was defined consistent with Society for Vascular Surgery Reporting Standards as: (1) successful creation of the fenestration through the deployed endograft fabric at the intended target vessel ostium; (2) successful deployment and patent flow through the bridging stent graft without kinking, thrombosis, or displacement; (3) absence of Type I endoleak or Type III endoleak on completion angiography; and (4) restoration of antegrade flow in the target supra-aortic branch without evidence of embolization or dissection of the target vessel.

### 2.9. Study Endpoints

To ensure conceptual clarity, the following endpoint hierarchy is adopted throughout this manuscript: (1) procedural technical success refers exclusively to intraoperative achievement of all four predefined components assessed on completion angiography; (2) 30-day clinical success is defined as freedom from all-cause mortality, freedom from reintervention, and freedom from major procedure-related complications within 30 days; and (3) follow-up imaging outcomes (endoleak, branch patency, reintervention) are reported as secondary endpoints distinct from intraoperative technical success. These categories are reported separately throughout the manuscript.

The primary endpoint was technical success rate, defined as the percentage of successful fenestration procedures relative to the total attempted. All four components of technical success were assessed at the time of the index procedure. Endoleaks identified on postoperative follow-up imaging are reported as secondary outcomes and are distinct from intraoperative completion angiography findings.

Secondary endpoints included: (1) freedom from reintervention at 30 days and beyond; (2) target vessel patency (LSA and LCCA, as applicable) on duplex ultrasound or CTA at each follow-up time point; (3) incidence of endoleak by type (Ia, Ib, II, III, IV) on completion angiography and follow-up imaging; (4) neurological event rate, defined as the composite of new permanent neurological deficit, stroke (ischemic or haemorrhagic confirmed on cross-sectional brain imaging), and TIA; (5) all-cause mortality; and (6) aortic-related mortality. Neurological outcomes were assessed clinically at each follow-up visit using a structured neurological examination and, in cases of clinical suspicion, brain imaging with CT or MRI.

This was a study with predefined peri-procedural and 30-day outcomes. Follow-up beyond 30 days was based on routine clinical visits and available institutional records and was not uniformly standardized across all patients.

### 2.10. Data Collection and Analysis

Patient demographics, procedural details, 30-day follow-up data, and intra- or postprocedural complications were systematically recorded in an Excel sheet (Microsoft^®^ Excel^®^, Microsoft 365 version 2606, Microsoft Corporation, Redmond, WA, USA) and analyzed for frequency distribution. Neurological outcomes were primarily assessed by clinical evaluation and imaging studies, as indicated. The data were analyzed to determine the feasibility and safety of the procedure using frequency tables.

Continuous variables are reported as mean ± standard deviation. Categorical variables are reported as absolute frequencies and percentages. Given the descriptive and exploratory nature of this single-arm study, no formal hypothesis testing or subgroup comparisons were performed; findings are reported as frequency distributions, consistent with the study’s primary aim of establishing safety and feasibility benchmarks.

## 3. Results

The patient cohort comprised 74 patients who underwent ISF-TEVAR, with a mean age of 70.6 (±9.3) years; 83.8% (*n* = 62) were male, and 16.2% (*n* = 12) were female. The demographic profile reflects a high cardiovascular comorbidity burden typical of this population, with male predominance consistent with reported sex distributions in thoracic aortic aneurysm and Type B dissection. Both elective and emergency cases were included; however, as procedural urgency was not systematically captured as a discrete variable in the institutional database, formal subgroup analysis by urgency status was not performed. ISF-TEVAR was combined with self-radiopaque-guided PMF in 28.4% (*n* = 21) of patients and with the chimney technique in 2.7% (*n* = 2). The distribution of major diagnoses, procedures performed, and devices used is presented in Table 1.

The primary endpoint of technical success was achieved in all 74 patients (100%; 95% CI: 95.2–100.0%). Specifically, fenestration was successfully created at the intended target vessel ostium in all cases; all bridging stent grafts were deployed with confirmed patency and without kinking, thrombosis, or displacement; completion angiography demonstrated the absence of Type I or Type III endoleak in all 74 cases; and antegrade flow in the target supra-aortic branch was confirmed in all cases without evidence of embolization or target vessel dissection. The Type Ia and Type III endoleaks reported in the follow-up data were identified on postoperative cross-sectional imaging and were not present on completion angiography, preserving the integrity of the predefined intraoperative technical success definition.

PMF was employed in cases requiring revascularization of the LCCA and/or innominate artery in addition to ISF for the LSA, reflecting greater aortic arch branch involvement. These cases were preoperatively planned based on CTA evaluation and did not represent intraoperative rescue decisions. Table 2 provides a descriptive breakdown of 30-day and post-30-day complications by procedural technique subgroup. Given the small event numbers within each subgroup, formal statistical comparisons were not performed; descriptive findings do not suggest a disproportionate complication burden in the combined ISF + PMF group relative to the ISF-alone group.

Complications were graded using the Clavien–Dindo classification: Grade I = deviation from the normal postoperative course requiring no intervention; Grade II = requiring pharmacological treatment; Grade IIIa = requiring reintervention without general anesthesia; Grade IIIb = requiring reintervention under general anesthesia; Grade IV = life-threatening complication requiring intensive care unit admission; Grade V = death. At 30 days, 30-day clinical success (freedom from all-cause mortality, reintervention, and procedure-related complications) was achieved in 87.8% (65/74; 95% CI: 78.2–94.3%) of patients. The overall 30-day complication rate was 12.2% (9/74; 95% CI: 5.7–21.8%). No retrograde type A dissection, intraoperative aortic rupture, conversion to open surgery, device infection, or access-site complication (access vessel injury, groin hematoma, pseudoaneurysm, or lymphocele) was recorded. All outcomes are presented in Table 3.

Endoleaks were detected in 7/74 patients (9.5%; 95% CI: 3.9–18.5%) on postoperative cross-sectional imaging; none were present on completion angiography. Type III endoleaks (*n* = 4 total: 3 within 30 days, 1 beyond 30 days) are attributed to incomplete apposition at the bridging stent–main body interface, a recognized complication of ISF techniques; two required reintervention, and two were managed conservatively. Type Ia endoleaks (*n* = 2: one within 30 days, one beyond 30 days) are consistent with proximal seal zone insufficiency; both underwent successful endovascular proximal cuff extension. Type II endoleaks (*n* = 3: two within 30 days, one beyond 30 days) were all managed conservatively; no sac enlargement was documented on serial surveillance imaging in any conservatively managed patient.

A total of six reinterventions were performed across the study period (8.1%; 95% CI: 3.0–16.8%), all of which were endovascular. Indications were subclavian branch thrombosis (*n* = 1, within 30 days), Type Ia endoleak (*n* = 2: one within 30 days, one beyond 30 days), Type III endoleak (*n* = 2, beyond 30 days), and chimney graft occlusion (*n* = 1, beyond 30 days). All reinterventions were technically successful with favorable clinical outcomes. No conversion to open surgery was required at any point. Given the limited sample size and the absence of standardized long-term follow-up, time-to-event analysis using the Kaplan–Meier method was not feasible. Details are presented in Table 4.

TIA, defined as a focal neurological deficit resolving completely within 24 h with no evidence of cerebral infarction on cross-sectional brain imaging, occurred in 1 patient (1.4%) within 30 days and in 2 additional patients (2.7%) beyond 30 days; all three recovered fully without residual neurological deficit or aortic-related complications. Confirmed stroke, defined as a new permanent neurological deficit with cerebral infarction on CT or MRI, occurred in 1 patient beyond 30 days and ultimately led to death at 13 months. No strokes were recorded within the 30-day follow-up window. Neurological events were assessed clinically at each follow-up visit; formal adjudication by an independent neurologist and routine postoperative diffusion-weighted MRI surveillance were not performed, and subclinical cerebral embolization events may therefore be underestimated.

No cases of spinal cord ischemia, paraplegia, paraparesis, or delayed spinal neurological deficit were recorded. Spinal cord protection measures included maintenance of mean arterial pressure >80 mmHg intraoperatively and in the immediate postoperative period, avoidance of hypotension, and preservation of LSA flow via ISF in all cases. Prophylactic cerebrospinal fluid drainage was not routinely employed unless the extent of aortic coverage or prior aortic repair was considered to confer high risk by the operating team.

In-hospital mortality was 0/74 (0%), and 30-day all-cause mortality was 0/74 (0%; 95% CI: 0.0–4.9%). Late all-cause mortality was 3/74 (4.1%) at the last available follow-up. One patient died at 60 days from myocardial infarction (cardiovascular mortality, considered likely non-procedure-related given the interval and baseline comorbidity burden); one at 13 months from confirmed ischemic stroke (neurological mortality; this patient had a documented permanent neurological deficit from beyond 30 days); and one at 9 months from aneurysm rupture (aorta-related mortality; the institutional record does not specify whether rupture occurred in the treated aortic segment or in a separate segment, and definitive attribution to device failure or disease progression cannot be confirmed from available data).

All 74 enrolled patients had complete 30-day follow-up data. Post-30-day follow-up was available for 63 patients (85.1%); 11 patients (14.9%) were lost to follow-up beyond 30 days. Follow-up duration and imaging availability were not uniformly recorded, precluding reporting of median follow-up or the number of patients with standardized imaging surveillance at predefined intervals. Late events reported beyond 30 days should therefore be interpreted with caution given incomplete and variable follow-up.

## 4. Discussion

Our patient cohort comprised predominantly elderly individuals (mean age ~70 years), consistent with the known epidemiology of thoracic aortic disease, where advanced age is a significant risk factor for both aneurysmal degeneration and dissection [19]. The present study primarily reports early clinical outcomes, with limited, non-standardized follow-up beyond 30 days, which limits conclusions regarding long-term durability.

The demographic profile of our cohort, predominantly elderly males with a high cardiovascular comorbidity burden, mirrors established epidemiological patterns of thoracic aortic disease and supports an endovascular-first approach in this population. The observed male predominance (83.8%) aligns with reported sex distributions in thoracic aortic aneurysm and Type B dissection, where ratios of 2–4:1 and approximately 3:1, respectively, have been described. Advanced age is additionally associated with increased baseline cerebrovascular vulnerability, reinforcing the importance of minimizing arch manipulation and embolic risk during supra-aortic interventions.

Device selection in this series was individualized according to lesion morphology, vessel diameter, arch anatomy, and operator preference over a six-year period during which device availability and institutional experience evolved [20]. The GORE^®^ Viabahn^®^ Endoprosthesis was preferentially used for bridging in angulated LSA anatomies due to its flexibility and low-profile delivery, reducing the risk of kinking at the arch–branch interface [21]. The Ankura™ TAA system represented the most frequently used thoracic endograft platform, reflecting its broad applicability across thoracic aortic pathologies [20]. Other devices, including Fluency™ Plus and Valiant^®^ systems, were used selectively based on anatomical requirements. Balloon-expandable stents were reserved for short, calcified, or high-resistance landing zones requiring greater radial force [22,23,24]. This heterogeneity reflects real-world practice and, although it introduces variability, procedural outcomes remained consistent across device combinations, supporting the broad applicability of ISF across platforms.

The aortic arch remains one of the most complex anatomical regions for endovascular repair due to its curvature, branch configuration, and embolic risk profile. TEVAR involving supra-aortic branches is associated with increased neurological risk [10,25], making preservation of cerebral and spinal perfusion a key procedural objective. While revascularization of the LSA and other arch branches is an established protective strategy [26], its execution is technically challenging in complex arch anatomy. For such cases, more complex endovascular techniques, such as ISF-TEVAR, may be necessary to preserve the blood flow.

In this context, ISF-TEVAR offers a pragmatic solution to extend the proximal landing zone while maintaining branch perfusion. In this series, the majority of cases involved arch aneurysms and dissections, consistent with previously reported distributions in ISF literature [13]. ISF enables intraoperative creation of a controlled fenestration within the deployed endograft, thereby overcoming limitations of fixed pre-manufactured designs and avoiding delays associated with custom device production.

Alternative techniques each carry distinct limitations. PMF preserves anatomical alignment and is associated with low endoleak rates [27,28], but remains highly operator-dependent and vulnerable to misalignment in tortuous anatomy [13,18]. The addition of radiopaque markers improves accuracy and reproducibility [18]. In this study, 66.2% of patients underwent ISF-TEVAR for the LSA, and with PMF in 28.4% of cases, demonstrating favorable outcomes. Chimney techniques offer rapid applicability in urgent settings and maintain branch patency by parallel grafting; however, they are associated with gutter-related endoleaks and variable durability [17]. A 30-day mortality rate of 1.2% and graft occlusion in 1.7% with chimney TEVAR for various aortic arch pathologies in participants with a mean age of 56 years has been reported [17]. In another study, although custom-made fenestrated TEVAR demonstrated no complications, chimney-TEVAR had a 20% reintervention rate [29]. Approximately 3% of patients in our study (*n* = 2) underwent chimney-TEVAR to preserve the LSA, with one patient demonstrating graft occlusion. In this context, ISF offers a balanced alternative by combining procedural immediacy with intraoperative adaptability and the avoidance of predeployment geometric constraints.

Compared with open arch replacement, ISF-TEVAR offers a substantially reduced perioperative mortality risk, particularly in elderly patients and those with significant comorbidities. Open total arch replacement is associated with in-hospital mortality rates and carries significant risks of stroke, cognitive decline, and prolonged cardiopulmonary bypass. Hybrid debranching, combining open cervical vessel transposition with TEVAR, reduces procedural complexity but still carries the risks of cervical surgery, cranial nerve injury, and wound complications. ISF-TEVAR avoids cervical dissection entirely, making it particularly attractive for high-surgical-risk patients. Off-the-shelf branched devices are increasingly available but remain subject to anatomical sizing constraints and, in most jurisdictions, require on-label anatomical eligibility that may exclude a proportion of patients with complex or urgent presentations. Custom-manufactured fenestrated/branched devices, while anatomically optimized, require manufacturing lead times that preclude their use in urgent scenarios, a clinical context in which ISF-TEVAR may offer its greatest advantage. The present series reinforces ISF-TEVAR as a pragmatic and reproducible option across the spectrum of aortic arch pathologies in experienced centers.

Neurological complications remain a major concern in TEVAR due to embolic phenomena and altered cerebral perfusion [10,25]. Stroke mechanisms include embolization of aortic arch atheroma and hypoperfusion of vertebral and carotid territories, while spinal cord ischemia relates to interruption of segmental arterial inflow [30]. Although reported rates are lower than open repair (2.5–5%), LSA coverage without revascularization remains a recognized risk factor. ISF-TEVAR may mitigate this risk by enabling routine preservation of supra-aortic branches, particularly the LSA, thereby supporting posterior circulation perfusion [12,30].

In this study, early outcomes demonstrated that a high proportion of patients remained free of complications at 30 days, suggesting good procedural tolerability of ISF-TEVAR. Technical success was confirmed in all cases on completion angiography, defined by successful fenestration, patent bridging stent flow, and absence of intraoperative Type I or III endoleak. Importantly, endoleaks detected during follow-up were identified on postoperative imaging and were not present intraoperatively, and therefore do not affect the reported technical success rate. These findings are consistent with previously reported favorable outcomes of ISF-based approaches [31].

ISF-TEVAR achieved a high rate of uncomplicated perioperative outcomes, reinforcing its feasibility in both elective and urgent settings. Although complications were observed in a minority of cases, overall early mortality remained low, supporting its safety profile in appropriately selected patients. The observed outcomes are consistent with previously reported ISF-TEVAR series demonstrating low perioperative mortality and acceptable complication rates [13,18,32].

This study adds to the existing literature in several ways. It represents a relatively large single-center experience evaluating ISF across heterogeneous aortic arch pathologies in both elective and emergency settings. Unlike prior studies focusing on single devices or isolated techniques, this series reflects a real-world, multimodality practice incorporating ISF, PMF, chimney techniques, and multiple endograft platforms. The consistently high technical success rate supports the reproducibility of ISF in experienced centers and reinforces its role as a flexible alternative to custom-manufactured branched systems, particularly in time-sensitive clinical scenarios.

Future research should focus on multicenter prospective registries with standardized imaging protocols to allow robust comparison across techniques and patient subgroups. Long-term follow-up is essential to evaluate the durability of the fenestration–bridging stent interface, particularly with respect to late Type III endoleak, branch stenosis, and reintervention rates, which remain underreported in the current literature. Emerging technologies, including image fusion, navigation systems, and augmented fluoroscopic guidance, may improve fenestration precision and reduce procedural variability. Comparative studies between ISF-TEVAR, PMF, chimney techniques, and commercially manufactured branched devices are also required to establish evidence-based treatment algorithms. Additionally, cost-effectiveness analyses will be important for defining the broader role of ISF in resource-constrained settings.

## 5. Limitations

This prospective single-center observational cohort study may have limited generalizability due to potential selection bias, convenience sampling, and a relatively small cohort size, resulting in reduced statistical power to detect subtle differences and rare but clinically important complications such as retrograde type A dissection, device migration, or late branch fracture. The high technical success rate and low complication rate may partly reflect operator expertise bias, as both surgeons had extensive prior experience in complex endovascular aortic surgery; therefore, reproducibility in lower-volume or less experienced centers remains uncertain. Furthermore, the heterogeneity of underlying aortic pathology (aneurysm, dissection, penetrating aortic ulcer, and intramural hematoma) may have influenced outcomes in ways that cannot be controlled in a descriptive single-arm series.

The absence of a control group limits comparative assessment against alternative arch repair strategies. In addition, procedural urgency was not systematically recorded as a categorical variable in the prospective database, precluding precise reporting and stratified analysis of elective versus emergency cases. As emergency aortic interventions may independently influence perioperative morbidity, neurological risk, technical complexity, and follow-up completeness, this represents a meaningful limitation of the current dataset. Future prospective multicenter studies should incorporate procedural urgency as a predefined stratification variable from the point of data collection.

The relatively short and incompletely standardized follow-up limits assessment of long-term durability, late complications, reintervention, endoleaks, mortality, and target vessel patency. Median follow-up duration, imaging surveillance rates, and systematic post-30-day patency data could not be consistently reported because follow-up protocols and imaging schedules were not uniformly maintained. Stent heterogeneity may also have introduced unadjusted device-related variability, although the findings remained broadly consistent with existing literature.

A further limitation is the absence of standardized, time-stratified target-vessel patency surveillance. Primary, assisted primary, and secondary patency rates at predefined intervals (30 days, 6 months, and 12 months) could not be calculated because of non-uniform imaging follow-up and the absence of systematic patency surveillance beyond 30 days. Available data confirmed two branch occlusion events (one subclavian thrombosis and one chimney graft occlusion), both successfully managed with reintervention; no additional branch failures were clinically identified. Future studies should incorporate prospective, standardized duplex ultrasound and CTA surveillance to enable robust patency reporting and late durability assessment.

Neurological outcomes in this series were assessed using structured clinical evaluation and clinically indicated brain imaging only; routine postoperative diffusion-weighted MRI surveillance was not performed. Given that subclinical cerebral embolization is a recognized consequence of endovascular aortic arch instrumentation, the true neurological burden in this cohort may have been underestimated. In addition, independent adjudication of neurological events, endoleaks, and deaths was not performed, as all outcomes were assessed by the treating team, introducing the possibility of ascertainment bias.

Several procedural and perioperative variables were not systematically recorded in the prospective database, including procedure duration, fluoroscopy time, contrast volume, intensive care unit length of stay, hospital length of stay, renal outcomes, contrast-induced nephropathy, and acute kidney injury classified by standardized criteria such as Acute Kidney Injury Network or Kidney Disease: Improving Global Outcomes. Consequently, a comprehensive assessment of procedural efficiency, resource utilization, and renal safety could not be performed. Future studies should incorporate these parameters as mandatory predefined secondary endpoints and standardized data fields.

## 6. Conclusions

In this prospective single-center observational cohort study, ISF-TEVAR was associated with a 100% technical success rate and an 87.8% 30-day clinical success rate (freedom from mortality, reintervention, and procedure-related complications), supporting its feasibility as a minimally invasive strategy for the management of complex aortic arch pathologies requiring supra-aortic branch preservation. The combined use of ISF with self-radiopaque-marker-guided PMF further extends its applicability to multi-vessel arch revascularization, potentially avoiding the need for surgical debranching, open arch replacement, or custom-manufactured devices with prolonged manufacturing lead times in selected cases. This is a particularly meaningful advantage in elderly or high-surgical-risk patients for whom open repair carries prohibitive morbidity.

Beyond favorable perioperative outcomes, this series also reported a low incidence of endoleaks and sustained branch artery patency. This is consistent with the acceptable hemodynamic performance of the technique in this cohort. ISF-TEVAR appears to be a feasible and safe endovascular option for selected aortic arch pathologies requiring supra-aortic branch preservation when performed in experienced centers. The favorable perioperative outcomes and low incidence of endoleaks observed in this series are encouraging. However, given the single-center, nonrandomized design and limited standardized follow-up beyond 30 days, broader conclusions regarding its role as a primary revascularization strategy are premature. Larger multicenter studies with standardized long-term imaging follow-up are warranted to confirm durability and define its position relative to alternative arch revascularization strategies.

## Figures and Tables

**Table 1:** Distribution of major diagnoses of patients, procedures performed, and devices used.

Major Diagnosis	*n*	Percentage
Type B aortic dissection	20	27.0%
Descending thoracic aortic aneurysm	37	50.0%
Penetrating aortic ulcer	8	10.8%
Aortic arch aneurysm	6	8.1%
Intramural hematoma	2	2.7%
Descending aortic pseudoaneurysm	1	1.4%
Procedures	*n*	Percentage
TEVAR + ISF for the LSA	49	66.2%
TEVAR + ISF for the LSA+ PMF for the LCCA	18	24.3%
TEVAR + ISF for the LSA + left carotid	3	4.1%
TEVAR + ISF for the LSA + chimney	2	2.7%
TEVAR + ISF for the LSA + PMF for the LCCA and IA	2	2.7%
Devices used	*n*	Percentage
Ankura^™^ TAA Stent Graft System	28	37.8%
GORE^®^ Viabahn^®^ Endoprosthesis	25	33.8%
Boston Scientific Express^™^ LD Iliac and Biliary Stent System	20	27.0%
Bard Fluency^™^ Plus Endovascular Stent Graft	5	6.8%
Dynamic balloon expandable stent, Biotronik	1	1.4%
Medtronic Valiant^™^ thoracic stent graft	1	1.4%

IA: Innominate artery; ISF: In situ fenestration; LCCA: Left common carotid artery; LSA: Left subclavian artery; PMF: Physician-modified fenestration; TEVAR: Thoracic endovascular aortic repair.

**Table 2:** Descriptive complications by procedural technique subgroup.

Technique	*n* (%)	30-Day Complications	Post-30-Day Complications
ISF alone (TEVAR + ISF for LSA)	49 (66.2%)	1 TIA; 1 subclavian thrombosis; 1 Type Ia endoleak; 3 Type III endoleak	1 Type Ia endoleak (reintervention); 1 Type Ib endoleak (conservative); 2 TIA (full recovery)
ISF + PMF (LCCA ± IA)	21 (28.4%)	1 Type II endoleak (LSA origin, conservative)	1 permanent neurological deficit (death at 13 months); 1 Type II endoleak (stable)
ISF + chimney	2 (2.7%)	None	1 chimney graft occlusion (reintervention)
ISF + PMF (LCCA + IA, multivessel)	2 (2.7%)	None	None

All values represent unique patients. Event numbers within subgroups are small; only descriptive presentation. IA: innominate artery; ISF: in situ fenestration; LCCA: left common carotid artery; LSA: left subclavian artery; PMF: physician-modified fenestration; TIA: transient ischemic attack.

**Table 3:** Consolidated follow-up outcomes: all values represent unique patients (*n* = 74).

Event	Patients (*n*)	Timing	Management	Clinical Consequence	Final Outcome
30-day outcomes					
No complications	65	≤30 days	—	—	Uneventful
Subclavian branch thrombosis	1	≤30 days	Reintervention	Branch occlusion	Resolved
TIA	1	≤30 days	Conservative	Transient neurological deficit	Full recovery
Type Ia endoleak	1	Postop CTA; not on completion angiography	Reintervention	Endoleak	Resolved
Type II endoleak	2	Postop CTA; not on completion angiography	Conservative	Endoleak	Stable; no sac expansion
Type II endoleak (from LSA)	1	Postop CTA; not on completion angiography	Conservative	Endoleak	Stable on surveillance
Type III endoleak	3	Postop CTA; not on completion angiography	2 reintervention; 1 conservative	Endoleak	Resolved (2); Stable (1)
Post-30-day outcomes					
No complications	52 *	>30 days	—	—	Uneventful
Chimney graft occlusion	1	>30 days	Reintervention	Branch occlusion	Resolved
Type Ia endoleak	1	>30 days	Reintervention	Endoleak	Resolved
Type Ib endoleak	1	>30 days	Conservative	Endoleak	Stable
Type II endoleak (from LSA)	1	>30 days	Conservative	Endoleak	Stable on surveillance
Type III endoleak	1	>30 days	Conservative	Endoleak	Stable
TIA	2	>30 days	Conservative	Transient neurological deficit	Full recovery
Permanent neurological deficit	1	>30 days	Supportive	Persistent deficit	Death at 13 months
Death (aneurysm rupture)	1	9 months	—	Fatal	—
Death (ischemic stroke)	1	13 months	—	Fatal	—
Death (myocardial infarction)	1	60 days post-procedure	—	Fatal	—

* Of 63 patients with available post-30-day follow-up data. All percentages calculated with *n* = 74 as the denominator unless otherwise stated. Some patients experienced more than one complication; all values represent unique patients per event type. TIA: transient ischemic attack; LSA: left subclavian artery; CTA: computed tomography angiography. All complications are classified by severity: Grade I = deviation from normal postoperative course requiring no intervention; Grade II = requiring pharmacological treatment; Grade IIIa = requiring reintervention without general anesthesia; Grade IIIb = requiring reintervention under general anesthesia; Grade IV = life-threatening complication requiring Intensive Care Unit; Grade V = death.

**Table 4:** Reintervention summary.

Reintervention	Timing	Indication	Technique Subgroup	Type of Reintervention	Technical Outcome	Clinical Outcome
1	≤30 days	Subclavian branch thrombosis	ISF alone	Endovascular recanalization/restenting	Successful	Resolved
2	≤30 days	Type Ia endoleak	ISF alone	Endovascular proximal cuff extension	Successful	Resolved
3	>30 days	Type Ia endoleak	ISF alone	Endovascular proximal cuff extension	Successful	Resolved
4	>30 days	Type III endoleak	ISF alone	Bridging stent revision/relining	Successful	Resolved
5	>30 days	Type III endoleak	ISF alone	Bridging stent revision/relining	Successful	Resolved
6	>30 days	Chimney graft occlusion	ISF + chimney	Endovascular reintervention	Successful	Resolved

All reinterventions were endovascular. No conversion to open surgery was required. Zone 2 was the treated arch zone in all cases. ISF: in situ fenestration.

## Data Availability

The datasets generated and analyzed during the current study are available from the corresponding author upon reasonable request.

## Abbreviations

The following abbreviations are used in this manuscript:

CT	Computed tomography
EVAR	Endovascular aneurysm repair
IA	Innominate artery
ISF	In situ fenestration
LCCA	Left common carotid artery
LSA	Left subclavian artery
PMF	Physician-modified fenestration
STEP	The Stroke from Thoracic Endovascular Procedures
TEVAR	Thoracic endovascular aortic repair
TIA	Transient ischemic attack
